# Oregano Essential Oils Mediated Intestinal Microbiota and Metabolites and Improved Growth Performance and Intestinal Barrier Function in Sheep

**DOI:** 10.3389/fimmu.2022.908015

**Published:** 2022-07-12

**Authors:** Li Jia, Jianping Wu, Yu Lei, Fanyun Kong, Rui Zhang, Jianxiang Sun, Liao Wang, Zemin Li, Jinping Shi, Ying Wang, Yubing Wei, Ke Zhang, Zhaomin Lei

**Affiliations:** ^1^ College of Animal Science and Technology, Gansu Agricultural University, Lanzhou, China; ^2^ Institute of Rural Development, Northwest Normal University, Lanzhou, China; ^3^ Key Laboratory of Animal Genetics, Breeding and Reproduction of Shanxi Province, College of Animal Science and Technology, Northwest A&F University, Yangling, China; ^4^ The Animal Husbandry and Veterinary Station in Pingshan Lake Mongolian Township of Ganzhou District, Zhangye, China

**Keywords:** oregano essential oil, ileal microbiota, metabolic profiles, mucosal immunity, *Lactobacillus reuteri*

## Abstract

With the increased demand for safe and sustainable alternatives to growth promoting antibiotics in the livestock industry, oregano essential oils (OEO) and *Lactobacillus reuteri* (LR) have been examined as alternatives to antibiotics for growth promotion and to improve animal health and performance. However, the mechanism underlying the OEO and LR mediation of sheep growth remains unknown. In this study, 16S rRNA gene sequencing and untargeted metabolomics were used to determine the role of the gut microbiota in the growth improvements observed. The potential modulating roles of intestinal microbial metabolites of OEO and LR to intestinal health were systematically explored as well. It was observed that both OEO and LR had greater average daily gain (ADG) and lower F/G ratio. Furthermore, OEO also appeared to have produced a greater amylase enzyme activity and mucin gene expression in the jejunal mucosa. It was also observed that OEO reduced serum IL-2 and TNF-β as well as mRNA levels of *NF-κB p65*, toll-like receptor-4 (*TLR-4*), and *IL-6* in the jejunal mucosa. Moreover, dietary OEO supplementation increased the abundances of *Ruminococcus*, *Bifidobacterium* and *Enterococcus*, while the relative abundances of *Succiniclasticum*, *Marvinbryantia* and *Streptococcus* were enriched in LR group. Spearman’s correlation analysis revealed that the abundances of *Bifidobacterium*, *Ruminococcus* and *Enterococcus* were positively correlated with the mRNA expression of mucins. Moreover, the relative abundance of *Enterococcus* was positively correlated with amylase activity. Metabolomics analysis indicated that OEO and LR increased the levels of indole acetaldehyde and indole-3-acetic acid through the tryptophan metabolism pathway. It was observed that LR also decreased the inflammatory metabolites including tryptamine and 5-hydroxyindole-3-acetic acid. Collectively, these results suggested that OEO exerted a beneficial effect on growth performance and the mucosal barrier, affected tryptophan metabolism and improved the intestinal microbiota of sheep.

## Introduction

The intestine is regarded as the first line of defense against food-born pathogens, and is the predominant organ involved in digestion and nutrient absorption (1). The mammalian intestine harbors a vast microbiota that plays a role in maintaining the intestinal barrier, immune functions and metabolic changes (2, 3). It is well established in the literature that intestinal microbiota aid in the digestion of food, and metabolizing many otherwise non-digestible dietary components into useful forms for the host as well as producing metabolites to regulate host health and immune defenses (4). Consequently, manipulation of intestinal microbiota and its metabolites by dietary modulation may be a potentially effective approach to improve animal health. Monensin (MON), an ionophore antibiotic, has a potential to modulate ruminal fermentation and improve animal performance (5, 6) However, some European countries have banned the use of antibiotic feed additives due to the fact that they have been demonstrated to produce antibiotic resistance. As a result, research for alternatives like essential oils, organic acids, probiotics and prebiotics is critical (7–9).

Oregano essential oils (OEO) extracted from plants have attracted considerable attention due to their health-promoting properties and the resulting potential in animal production. Carvacrol and thymol are the primary components of OEO. Carvacrol and thymol, both phenolic compounds, have a hydroxyl group that destroys bacterial cell membranes causing leakage of ions and molecules, thus exerting antibacterial activity (10, 11). It has been shown that dietary supplementation with 15 mg/kg OEO increased the average daily growth, ileal villus height, and lactic acid bacteria, while reducing coliforms in broiler chickens (12). Recent work demonstrated that OEO reduced the levels of serum cortisol and norepinephrine, therefore relieving stress and improving antioxidative activity caused by transportation stress in piglets (13). Furthermore, OEO may protect intestinal integrity through the promotion of antimicrobial peptide synthesis and upregulating the relative expression of claudin 1 (*CLDN1*) and mucin 2 (*MUC2*) (14). Emerging evidence also suggests the modulation of intestinal microbial composition acts as a crucial mechanism through which OEO may enhance the activity of the digestive enzyme chymotrypsin, improve gut morphology and epithelial barrier functions in addition to modulating the mucosal immune status of late-phase laying hens (15). It has also been demonstrated that OEO could increase the relative abundance of *Lactobacillus* and decrease the relative abundance of *Enterobacteriaceae* in piglets, suggesting that OEO could modulate the gut microbial communities with significant effects on piglet performance (16). Thus, OEO in swine and poultry appears to alter microbial communities for improved digestive health and growth efficiency. However, no studies on OEO modulation of intestinal microbiota and metabolite levels to effect ruminant growth performance and gut health have not been reported in the literature.

*Lactobacillus reuteri* (LR), which is a live, non-pathogenic bacteria, is an important member of the commensal gut bacteria. It has been reported that LR exhibits a high degree of adhesion, making it well suited to colonize the intestinal epithelium, thus preventing the attachment and multiplication of pathogenic microbes (17, 18). A recent study reported that LR had the ability to protect the intestinal mucosal barrier integrity through moderately activating the Wnt/β-catenin pathway to stimulate the proliferation of intestinal epithelia (19). However, the mechanisms used by different strains in the intestinal tract vary greatly. Our previous studies demonstrated that OEO and LR improved the growth performance, slaughter performance, and meat quality of sheep (20). Therefore, whether and how OEO and probiotics differ in impact on physiology and community composition and their metabolites in animal hosts are worthy of further exploration.

Here, the ileal microbiota and metabolites produced in response to OEO or LR dietary supplementation in sheep were investigated. It was observed that OEO increased the average daily gain (ADG), amylase enzyme activity in ileal contents, and improved intestinal barrier function of sheep. Sequencing of the 16S rRNA gene provided insights into possible microbial causes of the structural and functional changes in the ileum after OEO supplementation. Finally, metabolites of ileal contents were identified using an untargeted metabolomic approach. Collectively, this study shed light on the underlying mechanisms of OEO supplementation on intestinal microbiota of sheep, providing support for the application of OEO as promising alternative to in-feed growth-promoting antibiotics.

## Materials and Methods

### Animals and Experimental Design

The experimental design and procedures were approved by the Institutional Animal Care and Use Committee of the Gansu Agricultural University under permit No.GSAU-Eth-AST-2022-034. A total of 36 crossbred (small-tailed Han × Hu) female lambs (3 months old, a mean body weight 20.11 ± 0.19 kg) were randomly divided into 4 groups (3 pens, 3 lambs/pen). The CON group was fed a basal diet, the MON group received a basal diet plus 0.5 g/head/day MON. The OEO group received a basal diet with 52 mg/head/day OEO, and the LR group received a basal diet supplemented with 10 g/head/day LR, based on the manufacturers’ recommendation. The MON was supplied by XABC Biotechnology Co. Ltd. (Xian, China) at a 90% concentration. The OEO used to supplement the diet (containing 1.3% OEO from Origanum vulgare subsp. hirtum plants) was obtained from Ralco Inc., Marshall, MN, USA with 98.7% natural feed grade inert carrier. Supplementation with 52 mg/head/day was based on previously reported studies (21). Lyophilized LR (2 × 10^9^ CFU/g) was obtained commercially. The nutrient composition of the basal diet is presented in **Table S1** (NRC, 2007). Feed was offered in equal amounts at 07:00 and 18:00 daily. The study period lasted 90 days. All animals were provided *ad libitum* access to feed and water.

### Slaughter and Sample Collection

At the 90th day of the trial period, the sheep were slaughtered after 12 h feed restriction. Five milliliters of blood were collected from the jugular vein before slaughter. Serum was collected and stored at -20°C. Jejunal and ileal contents were collected from the mid-jejunum and mid-ileum segments, which were then flushed with PBS. One section was snap-frozen in liquid nitrogen and then stored at -80°C until the time of analysis. Other sections of jejunum and ileum (approximately 2 cm) were fixed in 4% paraformaldehyde for histological analysis. Mucosal samples from the jejunum were scraped and frozen in liquid nitrogen and then stored at -80°C for analysis of mRNA expression.

### Serum Complements, Immunoglobulins and Cytokine Levels

Serum samples were thawed at 4°C and mixed thoroughly prior to analysis. Complement 3 and C4 were measured using a commercially available kit (Beijing sino-uk institute of Biological Technology, Beijing, China) and analyzed in a semi-automatic biochemical analyzer (A-6, Beijing, China). The concentrations of immunoglobulins (IgA, G, M), IL-1β, IL-2, IL-4, IL-6, TNF-α and TNF-β in serum were detected by ELISA using commercially available kits and according to the manufacturer’s instructions (Beijing sino-uk institute of Biological Technology, Beijing, China).

### Intestinal Morphology and Jejunal Goblet Cell Numbers

Intestinal samples were removed from the 4% paraformaldehyde fixative solution and embedded in paraffin. Each segment was sliced to a thickness of 3μm. Sections were stained with hematoxylin and eosin for analysis. Villus height (VH), villus width (VW), crypt depth (CD) and VH/CD ratios were measured. Ten of the most complete and well-oriented villi and their associated crypts from each segment were measured at 40 × magnification by light microscopy (Motic BA 210, Xiamen, China) coupled with an image analyzer (Image-Pro Plus 6.0, Media Cybernetics, Bethesda, MD, USA). Goblet cells were stained using Alcian Blue-periodic acid Schiff (AB-PAS) and counted on 5 morphologically similar villi per sheep.

### Digestive Enzyme Activity in Ileal Contents

The ileal contents collected above were homogenized thoroughly in a saline solution, and centrifuged at 2500 × g for 15 min at 4°C. The supernatants were then collected and immediately frozen at -80°C for use in later analyses. The amylase, lipase and trypsin enzyme activities in the samples were determined using standard kits according to the manufacturer’s instructions (Nanjing Jiancheng Bioengineering Institute, Nanjing, China).

### Quantitative Real-Time PCR (qRT-PCR) Analysis

Total RNA was isolated from the jejunal mucosa samples using Trizol reagent (TransGen Biotech Co., Ltd., Beijing, China) according to the manufacturer’s instructions. The concentration and quality of total RNA were detected using a Nanodrop 2000 spectrophotometer (Thermo Fisher Scientific Inc., Waltham, Massachusetts, USA). Optical density ratios (260/280 nm) between 1.8 and 2.0 indicated that the RNA was pure and suitable for use in further analyses. The resulting total RNA was used to generate cDNA using the Evo M-MLV RT Kit with gDNA Clean for qPCR II (AG11711, Accurate Biotechnology, Hunan). The cDNA was then used for amplification in a real-time PCR System (LightCycler480, Roche, Basel, Switzerland) using the SYBR Premix Pro Taq HS qPCR Kit (AG11701, Accurate Biotechnology, Hunan). The reaction mixture (20 μL) for qPCR contained 10 μL 2 × SYBR Green Pro Taq HS Premix, 0.8 μL each of the forward and reverse primers (10 μmol/L), 2 μL cDNA and 6.4 μL RNase free water. The following PCR thermocycling profile was used: 30 s at 95°C; and 40 cycles of 95°C for 30 s, 5 s at 60°C. GADPH was used as endogenous control, and the relative expression of genes was calculated using the 2^−△△^ *^Ct^* method (22). The gene specific primers were designed using Primer 3.0 (Applied Biosystems, Foster City, CA, USA) and synthesized by Zhongke Yutong (Shanxi, China). Primer sequences can be found in **Table S2**.

### 16S rRNA Gene Sequencing of the Ileal Microbiota

Total genomic DNA was extracted from 36 ileal content samples using the E.Z.N.A. stool DNA Kit (Omega Bio-tek, Norcross, GA) according to the manufacturer’s instructions. The concentration and quality of the extracted DNA were assessed by a Nanodrop 2000 spectrophotometer (Thermo Fisher Scientific Inc., Waltham, Massachusetts, USA), and 2% agarose gel electrophoresis, respectively. The V3-V4 region of the bacterial 16S rDNA was amplified using the primers 338F (5’-ACTCCTACGGGAGGCAGCAG-3’) and 806R (5’-GGACTACHVGGGTWTCTAAT-3’) in the ABI GeneAmp^®^ 9700 PCR System (Applied Biosystems Life Technologies, Foster City, CA, USA). The PCR thermocycling profile used was as follows: 95°C for 3 min, followed by 28 cycles at 95°C for 30 s, 55°C for 30 s and 72°C for 45 s, with a final extension at 72 °C for 10 min. Then, the amplicons were purified from 2% agarose gels using the AxyPrep DNA Gel Extraction Kit (Axygen Biosciences, Union City, CA, United States) according to the manufacturer’s instructions. The purified amplicons were then subjected to paired-end sequencing (2 × 300 bp) on the Illumina MiSeq PE300 platform (Illumina, San Diego, USA) according to the standard protocols at Majorbio Bio Technology Co. Ltd. (Shanghai, China). The raw reads were deposited into the NCBI Sequence Read Archive (SRA) database under the accession number PRJNA744748.

The raw paired-end sequences were demultiplexed, quality-filtered with fastp (version 0.19.6) and merged with FLASH (version 1.2.7) (23, 24). High-quality reads were selected using the DADA2 (25) plugin in QIIME2 (version 2020.2) under the recommended parameters to generate amplicon sequence variants (ASVs). The taxonomy of each 16S rRNA gene sequence was analyzed in the RDP Classifier algorithm against the Silva 138 database. A comparison threshold of 70% was used for the analysis.

### Metabolite Profiles of Ileal Contents

Approximately 50 mg of ileal content samples were processed with 500 μL methanol/water (ice-cold, 70%, v/v) containing 2-chlorophenylalanine (1 μg/mL, included as an internal standard) and then vortexed for 3 min, followed by sonication in ice water for 10min. After centrifugation at 12,000 × g for 10 min, the supernatants were transferred to autosampler vials for UPLC-MS/MS analysis (metware).

Two microliters of samples were analyzed by UPLC-MS/MS (UPLC, Shim-pack UFLC SHIMADZU CBM A system; MS, QTRAP^®^ System). In both ESI positive and negative modes, the solvent system was composed of A (0.04% acetic acid in water) and B (0.04% acetic acid in acetonitrile) and was run at a flow rate of 0.4 mL/min using the following elution gradient: 95:5 V/V at 0 min, 5:95 V/V at 11.0 min, 5:95 V/V at 12.0 min, 95:5 V/V at 12.1 min, 95:5 V/V at 14.0 min. The following ESI source conditions were utilized as follows: source temperature 500°C; ion spray voltage (IS) 5500 V or -4500 V; ion source gas I (GSI), gas II (GSII), and curtain gas (CUR) were set at 55, 60, and 25.0 psi, respectively; the collision gas (CAD) was high.

### Statistical Analysis

The results of growth performance, serum parameters, digestive enzyme data, histology, and relative gene expression were analyzed between two groups using a Student’s *t-test*. The analyses were performed using SPSS (version 20.0) and expressed as means ± SEM. Data differences were considered to be statistically significant at a value of *P* < 0.05 and statistically tendency at 0.05 ≤ *P* < 0.10.

For microbial community profiling, the alpha diversity analysis included richness estimator (ACE and Chao 1) and diversity indices (Shannon and Simpson) were calculated based on ASV-level bacterial taxa with a Wilcoxon rank-sum test. The results were presented as box-and-whisker plots using GraphPad Prism (version 8.0). Principal coordinates analysis (PCoA) based on binary-hamming distances and the analysis of similarities (ANOSIM) were performed to analyze the similarity or difference of the compositions of the bacterial communities. Taxa abundances at the phylum and genus levels were statistically compared among groups using python 2.7. Linear discriminant analysis (LDA) coupled with effect size (LEfSe) was performed to determine the microbial differences among treatments and LDA > 2.5. The Wilcoxon rank-sum test was used to identify differences in the most abundant genera between groups. False discovery rate (FDR) was used to correct *p*-values.

The Analyst 1.6.3 software was used to process mass spectrometric data. Orthogonal projections to latent structures-discriminate analysis (OPLS-DA) were used to determine metabolic differences between the two groups. The parameters of R^2^ and Q^2^ were used to evaluate the OPLS-DA model validity to avoid over-fitting. The fold change (FC) was calculated as the ratio of mean peak values between each of the two groups. The significantly different metabolites were selected based on the variable important in projection (VIP) ≥ 1.00 and FC ≥ 2 or ≤ 0.5 between the two groups. The differentially expressed metabolites were represented as the log of peak area using box plots. The Kyoto Encyclopedia of Genes and Genomes (KEGG) database was used for annotation of the results, as well as for enrichment and classification for identification of pathway enrichment patterns. The correlations between the ileum bacterial and digestive enzyme, inflammatory cytokines, and barrier functions related gene expression, and ileum microflora-related metabolites were analyzed using the Spearman’s correlation test in R (version 3.1.1).

## Result

### Growth Performance and Serum Parameters

**Figure 1:**
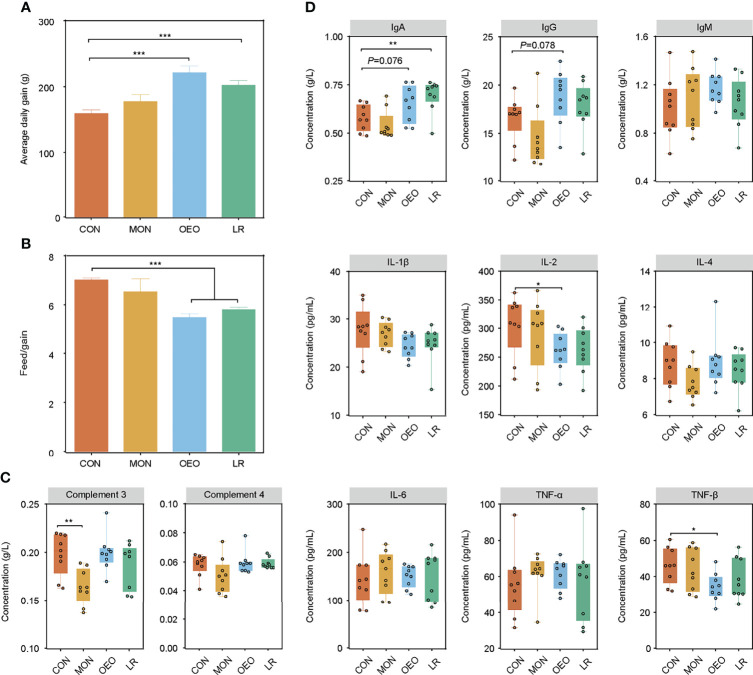
Effect of OEO and LR on the growth performance and serum parameters in sheep. **(A, B)** were results of growth performance of average daily gain (ADG), and feed/gain ratio (F/G). **(C, D)** The complements, immunoglobulins and pro-inflammatory cytokines contents in serum of sheep by different diet treatments. CON, a basal diet; MON, a basal diet plus 0.5 g/head/d monensin; OEO, a basal diet with 52 mg/head/d oregano essential oil; LR, a basal diet supplemented with 10 g/head/d *Lactobacillus reuteri.* Asterisks indicate significant difference between the trial group and the CON group (*0.01 < *P* ≤ 0.05; **0.001 < *P* ≤ 0.01; ***P ≤ 0.001). *IL*, interleukin; *TNF*, Tumor necrosis factor.

Compared to the CON group, OEO and LR supplementation significantly increased ADG and decreased the observed feed/gain (F/G) ratio (*P* < 0.001) (**Figures 1A, B**). Analysis of the serum immune related proteins showed that serum C3 was significantly decreased in the MON group (*P* < 0.01), OEO significantly decreased the IL-2 and TNF-β concentration (*P* < 0.05) and tended to increase IgA (*P* = 0.076) and IgG (*P* = 0.078) concentrations of serum, LR enhanced the IgA concentration (*P* < 0.01) relative to the CON group (**Figures 1C, D**). These results suggested that OEO improved growth performance and may exert anti-inflammatory effects.

### Intestinal Morphology, Jejunal Goblet Cell Numbers, and Digestive Enzyme Activity of Ileal Contents

**Figure 2:**
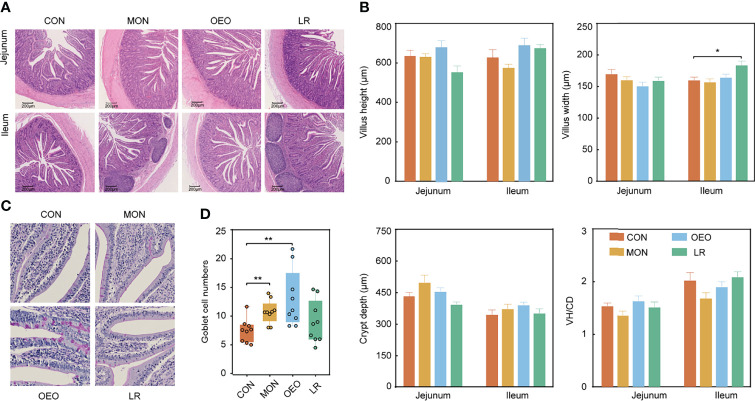
Effect of OEO and LR on intestinal morphology of jejunum and ileum in sheep **(A, B)** and jejunal goblet cell numbers **(C, D)**. VH/CD: villus height to crypt depth ratio. Results are means ± SEM (n = 9/group). CON, a basal diet; MON, a basal diet plus 0.5 g/head/d monensin; OEO, a basal diet with 52 mg/head/d oregano essential oil; LR, a basal diet supplemented with 10 g/head/d *Lactobacillus reuteri.* Asterisks indicate significant difference between the trial group and the CON group (*0.01 < *P* ≤ 0.05; **0.001 < *P* ≤ 0.01; ***P ≤ 0.001).

**Figure 3:**
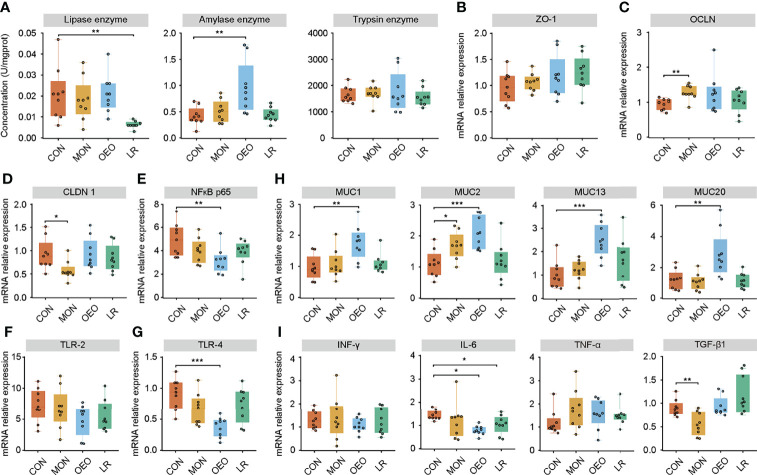
Effect of OEO and LR on the activities of digestive enzymes in ileal contents **(A)** and relative mRNA expression of genes in jejunal mucosa **(B–I)**. **(B–I)** were results of relative mRNA expression of genes related to tight junction proteins, mucin proteins and cytokines. Results are means ± SEM (n = 9/group). CON, a basal diet; MON, a basal diet plus 0.5 g/head/d monensin; OEO, a basal diet with 52 mg/head/d oregano essential oil; LR, a basal diet supplemented with 10 g/head/d *Lactobacillus reuteri.* Asterisks indicate significant difference between the trial group and the CON group (*0.01 < *P* ≤ 0.05; **0.001 < *P* ≤ 0.01; ***P ≤ 0.001). *ZO-1*, Zonula occludens 1; *OCLN*, Occluding; *CLDN1*, Claudin 1; *MUC*, Mucin; *NF-κB*, nuclear transcription factor-κB; *TLR*, Toll-like receptor; *IFN-γ*, Interferon-γ; *IL-6*, Interleukin-6; *TNF-α*, Tumor necrosis factor-α; *TGF-β1*, Transforming growth factor-β1.

Dietary supplementation with OEO alleviated the villus destruction and severe desquamation in the jejunum and ileum (**Figure 2A**). The VW of the ileum in the LR group was higher than that in the CON group (*P* < 0.05). However, no significant differences were observed for VH, CD, VH/CD among groups in the jejunum and ileum (*P* > 0.05) (**Figure 2B**). Inclusion of OEO and MON to the diets significantly increased the number of goblet cells in jejunal villi compared to the CON group (*P* < 0.01) (**Figures 2C, D**). In the ileum, the lipase activity was significantly decreased in the LR group (*P* < 0.01). Amylase activity was significantly increased in the OEO group (*P* < 0.01) when compared with CON group (**Figure 3A**). Overall, these results suggested that OEO supplementation provided benefits to intestinal morphology and digestive enzyme activity.

### mRNA Expression of Tight Junction Proteins, Mucins, and Cytokines in the Jejunal Mucosa

To assess the influence of OEO on intestinal barrier, the expression of tight junction proteins, mucins, and cytokines in the jejunal mucosa were measured by qRT-PCR. The mRNA expression of *OCLN* (*P* < 0.01) and *MUC2* (*P* < 0.05) increased, while the mRNA expression of *CLDN1* (*P* < 0.05) and *TGF-β1*(*P* < 0.01) decreased in the MON group compared to the CON group. Supplementation of OEO increased mRNA expression of *MUC1*, *MUC2*, *MUC13*, and *MUC20* (*P* < 0.01), but decreased the expression of nuclear factor kappa B (*NF-κB*) *p65* (*P* < 0.01), toll-like receptor-4 (*TLR-4*) (*P* < 0.001) and *IL-6* (*P* < 0.05), as compared with the CON group. The mRNA expression of *NF-κB p65* (*P* = 0.092) and *IL-6* (*P* < 0.05) decreased, and no significant differences were observed for the mRNA expression of *MUC1*, *MUC2*, *MUC13*, and *MUC20* in the LR group, as compared with the CON group (**Figures 3B–I**). These results indicated that OEO enhanced the mucosal barrier function.

### Microbial Composition in the Ileal Contents Using 16S rRNA Gene Sequencing

**Figure 4:**
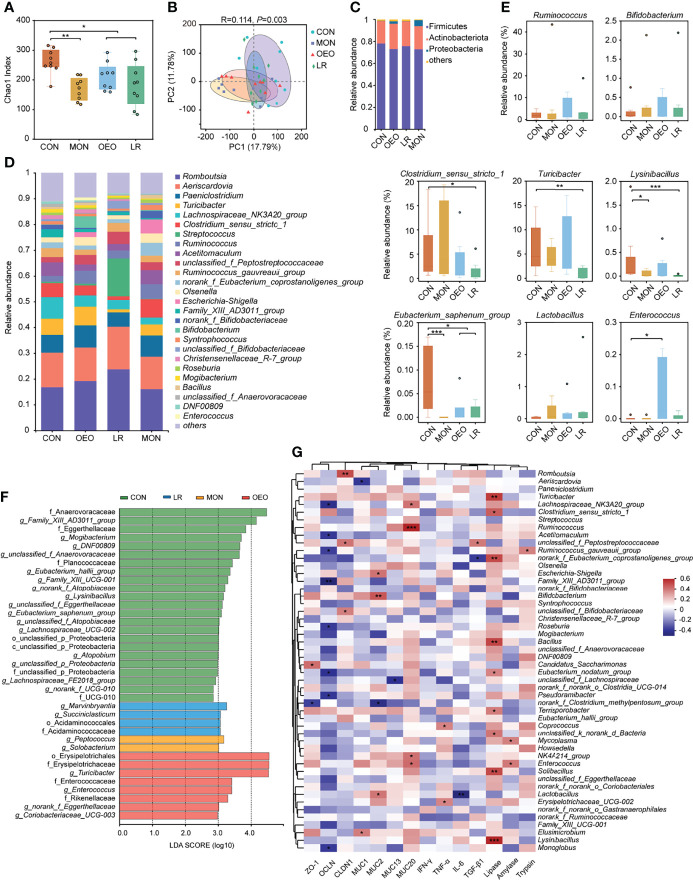
Microbial diversity of the sheep ileum. **(A)** Alpha diversity as presented by Chao1 index in the ileum of sheep among groups. **(B)** Principal coordinates analysis (PCoA) of bacterial communities in the ileal contents of sheep (based on the binary-hamming method). The composition of ileum microbiota of sheep at phylum **(C)** and genus **(D)** level. **(E)** Box plots showing the differences of taxonomic groups at the genus level. Data was presented as median and whiskers represented the Tukey. The statistical method was the Wilcoxon rank-sum test. **(F)** Total bacteria in the ileum of the sheep which make contributions to the difference at the phylum, class, order, family, and genus levels, as analyzed by the LDA effect size (LEfSe) method. **(G)** Heatmap of Spearman’s correlation analysis between the ileum bacterial, digestive enzyme, inflammatory cytokines and barrier functions related gene expression. Red represents a positive correlation, while blue represents a negative correlation. CON, a basal diet; MON, a basal diet plus 0.5 g/head/d monensin; OEO, a basal diet with 52 mg/head/d oregano essential oil; LR, a basal diet supplemented with 10 g/head/d *lactobacillus reuteri.* Asterisks indicate significant difference between the trial group and the CON group (*0.01 *< P* ≤ 0.05; **0.001 < *P* ≤ 0.01; ***P ≤ 0.001).

To further study whether OEO could affect the intestinal microbiota, the composition of the ileal microbiota was analyzed *via* sequencing of the 16S rRNA gene. A total of 1,965,934 V3–V4 16S rRNA effective sequences were obtained from the 36 samples, with an average of 54,609 sequences per sample. The α-diversity analysis revealed that ACE and Chao1 indices were significantly decreased in the trial group, and the Shannon index significantly decreased in the MON and LR groups. However, the Simpson index significantly increased in LR group (**Figure 4A**) (**Supplementary Figures S1A–C**). The PCoA plots using the binary-hamming method revealed that there were differences in microbiota between groups (ANOSIM, R = 0.114, *P* = 0.003) (**Figure 4B**).

At the phylum level, *Firmicutes*, *Actinobacteriota*, and *Proteobacteria* were the predominant bacteria (**Figure 4C**). At the genus level, the dominant genera were *Romboutsia* and *Aeriscardovia* (**Figure 4D**). According to the statistical analysis, OEO increased the relative abundance of *Ruminococcus* (4.98 ± 1.52 vs 2.20 ± 0.51), *Bifidobacterium* (4.47 ± 4.32 vs 0.13 ± 0.08), *Enterococcus* (*P* < 0.05), but decreased the relative abundance of *Eubacterium_saphenum_group* relative to CON (*P* < 0.05). Conversely, LR increased the relative abundance of *Streptococcus* (14.64 ± 9.78 vs 0.57 ± 0.31) and *Lactobacillus* (0.35 ± 0.83 vs 0.02 ± 0.03), but decreased the relative abundance of *Turicibacter* (*P* < 0.01), *Lysinibacillus* (*P* < 0.001), *Clostridium_sensu_stricto_1*, and *Eubacterium_saphenum_group* (*P* < 0.05). It was observed that MON decreased the relative abundance of *Lysinibacillus* (*P* < 0.05) and *Eubacterium_saphenum_group* (*P* < 0.05). Compared with the CON group, the relative abundances of *norank_f_norank_o_Saccharimonadales* was significantly higher in the MON group, whereas *Enterococcus* was significantly higher in the OEO group and *Marvinbryantia* and *Succiniclasticum* were significantly higher in the LR group (**Figure 4E**) (**Supplementary Figures S1D–F**).

The different bacteria from phylum to genus level that were specific between the trial and CON groups were identified by LEfSe analysis (**Figure 4F**). There were 23, 2, 8, 4 dominant taxa in ileal content samples of sheep fed CON, MON, OEO and LR diets, respectively. Anaerovoracaceae, *Family_XIII_AD3011_group*, *Mogibacterium*, *Family_XIII_UCG-001*, *Eubacterium_saphenum_group*, *unclassified_f_Anaerovoracaceae*, Eggerthellaceae, *DNF00809*, *unclassified_f_Eggerthellaceae*, Planococcaceae, *Lysinibacillus*, *Eubacterium_hallii_group*, *Lachnospiraceae_UCG-002*, *Lachnospiraceae_FE2018_group*, *Atopobium*, *norank_f_Atopobiaceae*, *unclassified_f_Atopobiaceae*, UCG-010 and *norank_f_UCG-010* were enriched in the CON group. *Peptococcus* and *Solobacterium* were enriched in the MON group, whereas Erysipelotrichales, Erysipelotrichaceae, *Turicibacter*, Enterococcaceae, *Enterococcus*, Rikenellaceae, *norank_f_Eggerthellaceae* and *Coriobacteriaceae_UCG-003* were enriched in the OEO group. *Marvinbryantia*, Acidaminococcales, Acidaminococcaceae and *Succiniclasticum* were enriched in the LR group. Collectively, these results demonstrated that multiple beneficial bacteria were significantly enriched in the OEO supplemented group.

### Correlation Analysis Between the Ileum Bacteria, Digestive Enzymes, Barrier Functions, and Gene Expression of Inflammatory Cytokines

A Spearman correlation analysis was employed to investigate the correlations among intestinal barrier functions, mucin gene expression, inflammatory cytokines, digestive enzyme activity, and the predominant microbial genera. In this study, the abundance of *Bifidobacterium* (*P* < 0.01) and *Lactobacillus* (*P* < 0.05) were positively correlated with *MUC2* expression, and *Lactobacillus* (*P* < 0.01) abundance was negatively correlated with *IL-6* expression. *Ruminococcus* (*P* < 0.001) and *Enterococcus* (*P* < 0.05) were positively correlated with *MUC20* expression. Moreover, the relative abundance of *Enterococcus* was positively correlated with amylase activity (*P* < 0.05). The lipase activity was positively correlated with *Turicibacter* (*P* < 0.01) and *Lysinibacillus* (*P* < 0.001) (**Figure 4G**). These results further indicated that the effects of OEO regulated intestinal microbiota may contribute to improve the intestinal function.

### Metabolite Profiles of Ileal Contents

**Figure 5:**
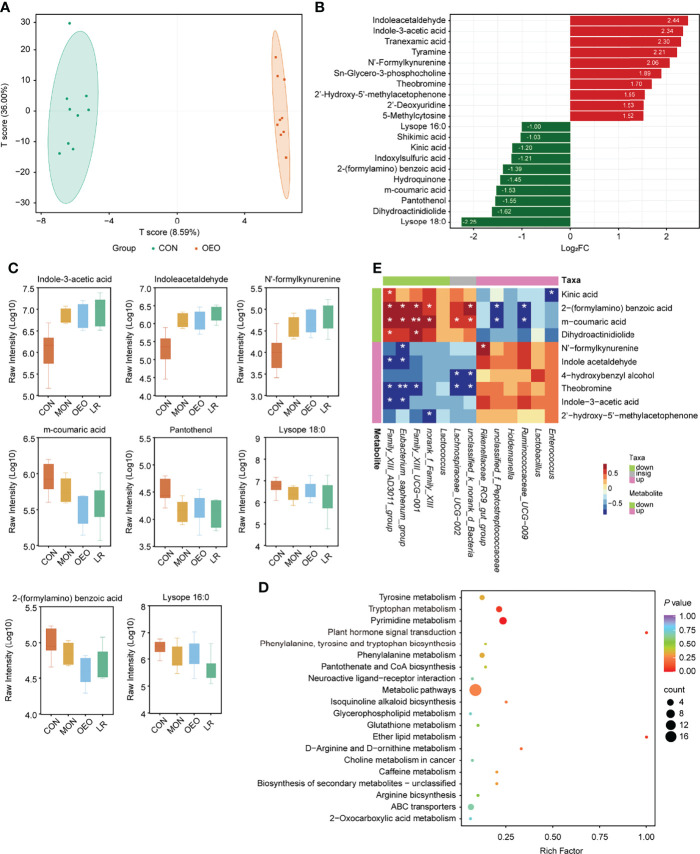
Results of ileum metabolites analysis. **(A)** OPLS-DA score plots showed significantly separated clusters between CON and OEO group. **(B)** Bar-chart showing the fold change of top 20 different metabolites. **(C)** Box-plot of each significantly different metabolite peak area (Log10 transformed) among treatments. Data was presented as median and whiskers represented the Tukey. **(D)** The KEGG functional enrichment analysis of differential metabolites (CON vs OEO). **(E)** Correlation analysis of microbiota and metabolites in the ileal contents between CON and OEO groups. Red represents a significant positive correlation (*P* < 0.05), while blue represents significantly negative correlation (*P* < 0.05). Asterisks indicate significant difference between the trial group and the CON group (*0.01 < *P* ≤ 0.05; **0.001 < *P* ≤0.01; ***P ≤ 0.001).

The UPLC-MS/MS platform was used to analyze the changes of ileal content metabolite profiles in sheep provided either standard or supplemented diets. A total of 465 metabolites were detected. The OPLS-DA indicated a clear separation between the CON and OEO groups (R^2^X = 0.590, R^2^Y = 0.996, Q^2^ = 0.567, **Figure 5A**), the CON and MON groups (R^2^X = 0.518, R^2^Y = 0.990, Q^2^ = 0.718, **Supplementary Figure S2A**), and the CON and LR groups (R^2^X = 0.650, R^2^Y = 0.995, Q^2^ = 0.795, **Supplementary Figure S2B**), which suggested that these groups had differential metabolites in the contents. In addition, the value of Q^2^ > 0.5 suggests that the OPLS-DA models were reliable.

The parameters of the variable importance in the project (VIP ≥ 1.00 and FC ≥ 2 or ≤ 0.5) were used to screen out differentially expressed metabolites (**Supplementary Table S3**, **Figures 5B,C**, **Supplementary Figures S2C–E**). When comparing the CON and OEO groups, 31 metabolites with significant differences were observed, 21 being up-regulated and 10 down-regulated. Levels of indole acetaldehyde (IAAD), indole-3-acetic acid (IAA) and N’-formylkynurenine (NFK) were significantly increased in response to OEO supplementation, while 2-(formylamino) benzoic acid was significantly decreased. When comparing the CON and MON groups, 31 metabolites with significant differences were observed, with 15 up-regulated and 16 down-regulated. Levels of taurochenodesoxycholic acid (TCDCA), spermidine, IAAD, IAA, and NFK were significantly increased by MON, while taurocholic acid (TCA) was significantly decreased. Between the CON and LR groups, 63 metabolites with significant differences were observed, with 24 being up-regulated and 39 down-regulated. Levels of IAAD and IAA were significantly increased by LR, while tryptamine and 5-hydroxyindole-3-acetic acid (5-HIAA) decreased significantly. Additionally, compared with the CON group, levels of lysope 16:0, lysope 18:0, and pantothenol were significantly decreased in all experimental groups.

Compared with the CON group, sheep receiving the OEO supplement mainly exhibited alterations in pyrimidine metabolism and tryptophan metabolism (**Figure 5D**), whereas the MON group exhibited a greater impact on 4 metabolic pathways including cholesterol metabolism, primary bile acid biosynthesis, bile secretion and tryptophan metabolism (**Supplementary Figure S2F**). The LR group was enriched in the pathway of tryptophan and phenylalanine metabolism (**Supplementary Figure S2G**). Thus, dietary supplementation with OEO may have important modulatory effects on tryptophan metabolism.

### Microbiota-Related Metabolites in the Ileum

The Spearman correlation coefficient was used to investigate the potential associations between the ileum microbiota and differential metabolites observed in the same animals (|R| > 0.6, *P* < 0.05). When a comparison was made between the CON and OEO groups, *Eubacterium_saphenum_group* was significantly positively correlated with 2-(formylamino) benzoic acid (R = 0.733, *P* = 0.033) and m-coumaric acid (R = 0.721, *P* = 0.034), but was negatively correlated with NFK (R = -0.673, *P* = 0.038), IAAD (R = -0.696, *P* = 0.035) and IAA (R = -0.735, *P* = 0.034). *Enterococcus* was negatively correlated with kinic acid (R = -0.709, *P* = 0.035, **Figure 5E**). Between the CON and MON groups, *Eubacterium_saphenum_group* was significantly negatively correlated with TCDCA (R = -0.718, *P* = 0.048) and spermidine (R = -0.776, *P* = 0.026), whereas *Bacteroides* was significantly positively correlated with NFK (R = 0.759, *P* = 0.029) and IAAD (R = 0.752, *P* = 0.030, **Supplementary Figure S2H**). Between the CON and LR groups, *Eubacterium_saphenum_group* was significantly positively correlated with lysope 16:0 (R = 0.667, *P* = 0.045) and pantothenol (R = 0.740, *P* = 0.024). *Lysinibacillus* was negatively correlated with IAAD (R = -0.770, *P* = 0.019), NFK (R = -0.785, *P* = 0.014) and IAA (R = -0.710, *P* = 0.036, **SupplementaryFigure S2I**).

## Discussion

There is an increasing interest in the usage of dietary OEO and LR for antibiotic-free livestock to improve growth, health status, immune responses, and modulate the gut microbiome (14, 26–29). As expected, the data presented here show that OEO and LR supplementation improved ADG and decreased F/G ratio, which was in accordance with previous studies (30, 31). However, comparison of the regulatory effects of OEO and LR on sheep has not yet been published.

Serum immunoglobulins are used to reflect humoral immunity. As potent inflammatory cytokines, IL-2 and TNF-β are involved in cellular immunity (32, 33). In this study, serum concentrations of IgA and IgG tended to increase, while IL-2 and TNF-β decreased in sheep fed a standard diet supplemented with OEO. Similarly, supplemental OEO and Co-lactate increased the concentrations of IgA, IgG, and IgM in lambs (34). Previous studies have shown that dietary essential oil supplementation leads to a reduction of inflammatory cytokines in the serum (IL-1β and TNF-α) (35, 36). The data presented here corroborate these other studies, demonstrating that dietary OEO supplementation might contribute to improvements in immune status of sheep. Furthermore, intestinal digestive enzyme activity plays an important role in the digestion and absorption of nutrients and production performance of animals. It has been reported that the addition of commercial EO stimulated the secretion of amylase enzyme, which was detected in the intestinal digesta of chickens (37). Previous studies indicated that feed supplemented with thymol and carvacrol improved the activities of intestinal digestive enzymes in broiler chickens (38). In the current study, sheep feed diets supplemented with OEO exhibited increased amylase activity in the contents of the small intestines. Thus, it could be postulated that the growth-promoting effects of OEO are likely dependent on the alterations in the intestinal microbiota and the associated changes in enzymatic activity. It was also found that the addition of LR decreased the activities of lipase enzyme. However, this observation contradicts those that were previously reported, and will require further study to confirm (39, 40).

In the current study, it was observed that OEO improved intestinal barrier integrity of the small intestine mucosa in the jejunum and ileum. Sheep fed diets supplemented with LR exhibited significant increases in VW in the ileum compared with control animals. Both LR and OEO supplementation had positive impacts on intestinal morphology (41, 42). Goblet cells can synthesize and produce mucus, such as MUC2, the predominant component of mucus, which appeared to maintain the intestinal surface mucus layer (43, 44). Indeed, *MUC2* gene expression was significantly increased in the ileum of sheep fed diets supplemented with MON or OEO, which is consistent with the observed increase in goblet cell numbers. The proteins encoded by the *ZO-1*, *OCLN*, and *CLDN1* genes are mainly constituents of intestinal tight junctions, which affect the paracellular barrier functions (45, 46). Animals in the MON group had upregulated mRNA expression of *OCLN*. It is generally accepted that intestinal inflammation is closely associated with the integrity of the intestinal barrier. As the members of typical pattern recognition receptors, toll-like receptors (TLRs) can mediate the recognition of microbial molecules to promote immune responses (47). TLR-4 can recognize lipopolysaccharides (LPS), which is a major component of the outer membrane of Gram-negative bacteria. It has proved that OEO can suppress the expression of *TLR-4*, *IL-1β*, *TNF-α* and *IFN-γ* through the TLR4-mediated signaling pathway (15). Besides, the activation of TLRs signaling stimulates the NF-ĸB signal pathway and then increases proinflammatory cytokine production including IL-1β, TNF-α, IL6, etc. (48, 49). A previous study reported that OEO downregulated the mRNA expression of *TNF-α* and *IL-6* in rats (50). In this study, OEO supplementation reduce gene expression of the *NF-κB p65*, *TLR-4* and pro-inflammatory cytokine *IL-6*, indicating that OEO could reduce the production of cytokines *via* suppressing inflammatory signaling pathway expression in the ileum. *Lactobacillus reuteri* NK33 could alleviate gut inflammation by suppressing the expression of *TNF-α* and *IL-6* through activation of the NF-κB signaling pathway (51). Similarly, we observed that LR reduced expression of *IL-6*. Collectively, OEO and LR supplementation exerted different effects on protecting the intestinal integrity. It was observed that OEO promoted mucin protein synthesis and reduced secretion of pro-inflammatory cytokines, while LR only appeared to inhibit expression of several pro-inflammatory cytokines.

To better clarify the effect of different additives in diets on intestinal microbiota, 16S rRNA analysis is used to evaluate the effects. In this study, the microbiota diversity and richness were significantly reduced by the experimental variables compared to that which was observed in control animals fed a standard diet. In line with previous studies, MON produced lower Chao1 and Shannon index values (52), and there were no differences in bacterial community composition when compared with that of animals supplemented with LR (53). The lower diversity in the rumen microbiota can increase feed efficiency (54). On the contrary, a previously published study demonstrated that EO-supplementation (62.5 mg/kg carvacrol and 7.5 mg/kg thymol) does not impact the alpha diversity of the colonic microbiota in piglets, although the microbial community structure was altered (55). The differences between the two studies might due to the different animal models examined.

In this study, the 3 experimental groups decreased the relative abundance of *Eubacterium_saphenum_group* in the ileum compared to those in the CON group. *Eubacterium_saphenum_group* has been described as a periodontal pathogen/pathobiont (56). Notably, the relative abundance of some beneficial bacteria such as *Bifidobacterium*, *Ruminococcus* and *Enterococcus* were also increased in the OEO group. Some species from *Enterococcus* such as *E. faecium* and *E. faecalis* and *Bifidobacterium* are known as natural probiotics on account of their multiple health-promoting effects in animal production (57–60). It has been reported that omnivorous beetles enriched with *Enterococcus* bacterium have the ability to more efficiently digest a greater volume of food (61). In addition, diets containing *Enterococcus* impart beneficial effects on increasing pupal weight and survival rate (62). Similarly, our study indicates that *Enterococcus* is positively correlated with amylase activity. *Bifidobacterium* has been demonstrated to inhibit pathogen colonization in the gut as a result of the bacteriocins it produces (63). A previous study has also reported that *Bifidobacterium* could strengthen intestinal mucosal immune barrier functions by stimulating colonic mucus growth (64). Similarly, this study indicated that *Bifidobacterium* abundance was positively correlated with *MUC2* expression. Therefore, these results indicate that the increased activity of digestive enzymes and the MUC2 secretion may be associated with intestinal microbial composition in OEO supplemented animals. The relative abundance of *Succiniclasticum*, *Marvinbryantia* and *Streptococcus* are significantly increased in the LR group. *Succiniclasticum* obtains metabolic energy exclusively by converting succinate to propionate (65). *Marvinbryantia* is believed to modulate the dysbiosis of the gut microbiota in mice with chronic colitis, and affects intestinal epithelial cell energy metabolism and butyrate production (66–68). *Streptococcus* (those belonging to the order Lactobacillales) are capable of producing SCFAs, which confer anti-inflammatory properties through regulating Tregs (69). In the present study, the abundance of *Lactobacillus* is negatively correlated with *IL-6* expression. *Lactobacillus* has been shown to improve the intestinal epithelial barrier function and has an anti-inflammatory effect in mice with colitis (70). Thus, we speculate that the amelioration of intestinal inflammation in LR supplemented sheep may be linked to changes in the intestinal microbiota and SCFA levels. The exact mechanism requires further evaluation to be determined.

It can be concluded that the considerable alterations in the ileal content metabolism are greatly influenced by different biological agents in diets as determined by our metabolomics analysis. In the present study, the concentrations of TCDCA and spermidine related to bile acid metabolism are increased by MON, while TCA is significantly decreased. Bile acids are synthesized from cholesterol in the liver, are important for efficient lipid absorption and cholesterol homeostasis (71). The anti-inflammatory and immuno-regulatory activities of TCDCA have been reported, while TCA is believed to stimulate intestinal bacteria by converting taurine and cholic acid to hydrogen sulfide and deoxycholic acid, a genotoxin and carcinogenic molecule (72, 73). Spermidine is important for maintaining tight junctions by upregulating expression of several tight junction proteins and by reducing DAO and D-lac levels (74). This notion is supported by the decrease in pathogenic bacteria and the increased expression of the tight junction protein *OCLN* in the present study. Furthermore, the tryptophan pathway is of particular interest because pathway analyses were enriched in animals receiving diets supplemented with OEO and LR. In the same groups, IAAD and IAA were significantly increased compared with those in the CON group. Tryptophan (Trp) is an essential amino acid, serving as a precursor for the synthesis of microbial and host metabolites (75). IAA and IAAD, microbial catabolites of tryptophan, regulate gut barrier function *via* the aryl hydrocarbon receptor (AhR) (76). Consistent with our research, LR exhibits the properties of metabolize tryptophan (L-Trp) to indole derivatives (77). In addition, the abundances of inflammatory metabolites including tryptamine and 5-HIAA are decreased in LR supplemented animals. It has been reported that 5-HIAA is positively associated with colitis (78). Tryptamine is produced by gut bacteria and can be converted to IAA by a combination of bacterial and mammalian enzymes (79, 80). Therefore, this alternation in tryptophan metabolism in the present study may have a positive effect on the growth and health of the sheep.

Combined with the microbial-metabolome analysis, the spearman correlation shows that *Eubacterium_saphenum_group* is positively correlated with 2-(formylamino) benzoic acid and m-coumaric acid, and is negatively correlated with TCDCA, spermidine, and IAAD. M-coumaric acid exerts inhibitory effects on cell growth and the proliferation of 3T3-L1 preadipocytes (81). We speculate that the tryptophan and bile acid metabolism in OEO/LR and MON trial groups, respectively, were related to the change in *Eubacterium_saphenum_group*.

## Conclusion

In conclusion, the data presented here demonstrate that dietary supplementation with oregano essential oils impacted the composition and metabolites of intestinal microbiota, promoted the enrichment of *Ruminococcus*, *Bifidobacterium*, and *Enterococcus*, and increased the levels of the metabolites indole-3-acetic acid and indole acetaldehyde. These changes in intestinal microbiota might contribute to increases amylase activity, thus improving growth performance and intestinal barrier function. Accordingly, these findings will provide insights into future application of alternative strategies for improving health and growth promotion in small ruminant species.

## Data Availability Statement

The sequencing datasets are available in the NCBI database under accession PRJNA744748.

## Ethics Statement

The animal study was reviewed and approved by the Institutional Animal Care and Use Committee of the Gansu Agricultural University under permit number No.GSAU-Eth-AST-2022-034.

## Author Contributions

LJ, ZLe, and JW designed the experiments. FK and YWe participated in the animal experiments. LJ, YL, FK, RZ, JSu, LW, ZL, JSh, and YWa helped to conduct the experiments and part of the laboratory work. LJ analyzed the data and wrote the manuscript. The manuscript was modified by ZLe and KZ. The authors read and approved the final manuscript.

## Funding

This research was financially supported by the China Agriculture Research System (CARS-38), Modern Agriculture Industrial System Project of Gansu Province (GARS-CS-1), Agro-scientific Research in the Public Interest (201503134), and the Agricultural special project of Gansu Province (GSSLCSX-2020-1).

## Conflict of Interest

The authors declare that the research was conducted in the absence of any commercial or financial relationships that could be construed as a potential conflict of interest.

## Publisher’s Note

All claims expressed in this article are solely those of the authors and do not necessarily represent those of their affiliated organizations, or those of the publisher, the editors and the reviewers. Any product that may be evaluated in this article, or claim that may be made by its manufacturer, is not guaranteed or endorsed by the publisher.

## Supplementary Material

Supplementary Table 1 Composition and nutrient levels of the basal diet.

Supplementary Table 2 Primers sequences used for real-time PCR.

Supplementary Table 3 Candidate ileal metabolites that differed between the CON and trial groups.

Supplementary Figure 1
**(A–C)** Alpha diversity as presented by ACE, Shannon, Simpson index in the ileal contents of sheep among groups. **(D–F)** Extended error bar plot showing the bacteria at the genus level that had significant differences between the CON and trial groups. Positive differences indicate greater abundance of bacteria in the CON group, while negative differences indicate greater abundance in the trial group.

Supplementary Figure 2
**(A, B)** OPLS-DA score plots showed significantly separated clusters between trial and CON group (CON vs MON, CON vs LR). **(C)** Box-plot of each significantly different metabolite peak area (Log10 transformed) among treatments. Data was presented as median and whiskers represented the Tukey. **(D, E)** Bar-chart showing the fold change of top 20 different metabolites (CON vs MON, CON vs LR). **(F, G)** The KEGG functional enrichment analysis of differential metabolites (CON vs MON, CON vs LR). **(H, I)** correlation analysis of microbiota and metabolites in the ileal contents (CON vs MON, CON vs LR). Red represents a significant positive correlation (*P* < 0.05), while blue represents significantly negative correlation (*P* < 0.05). Asterisks indicate significant difference between the trial and CON group (*0.01 < *P* ≤ 0.05; **0.001 < *P* ≤ 0.01; ****P* ≤ 0.001).
