# Supplementary material for: Oregano Essential Oils Mediated Intestinal Microbiota and Metabolites and Improved Growth Performance and Intestinal Barrier Function in Sheep
Source: Front Immunol. 2022 Jul 12;13:908015. doi: 10.3389/fimmu.2022.908015 (PMC9314563; doi:10.3389/fimmu.2022.908015)
Supplement: Supplementary Table 1 — Composition and nutrient levels of the basal diet. [file DataSheet_1.docx]

Supplementary Material


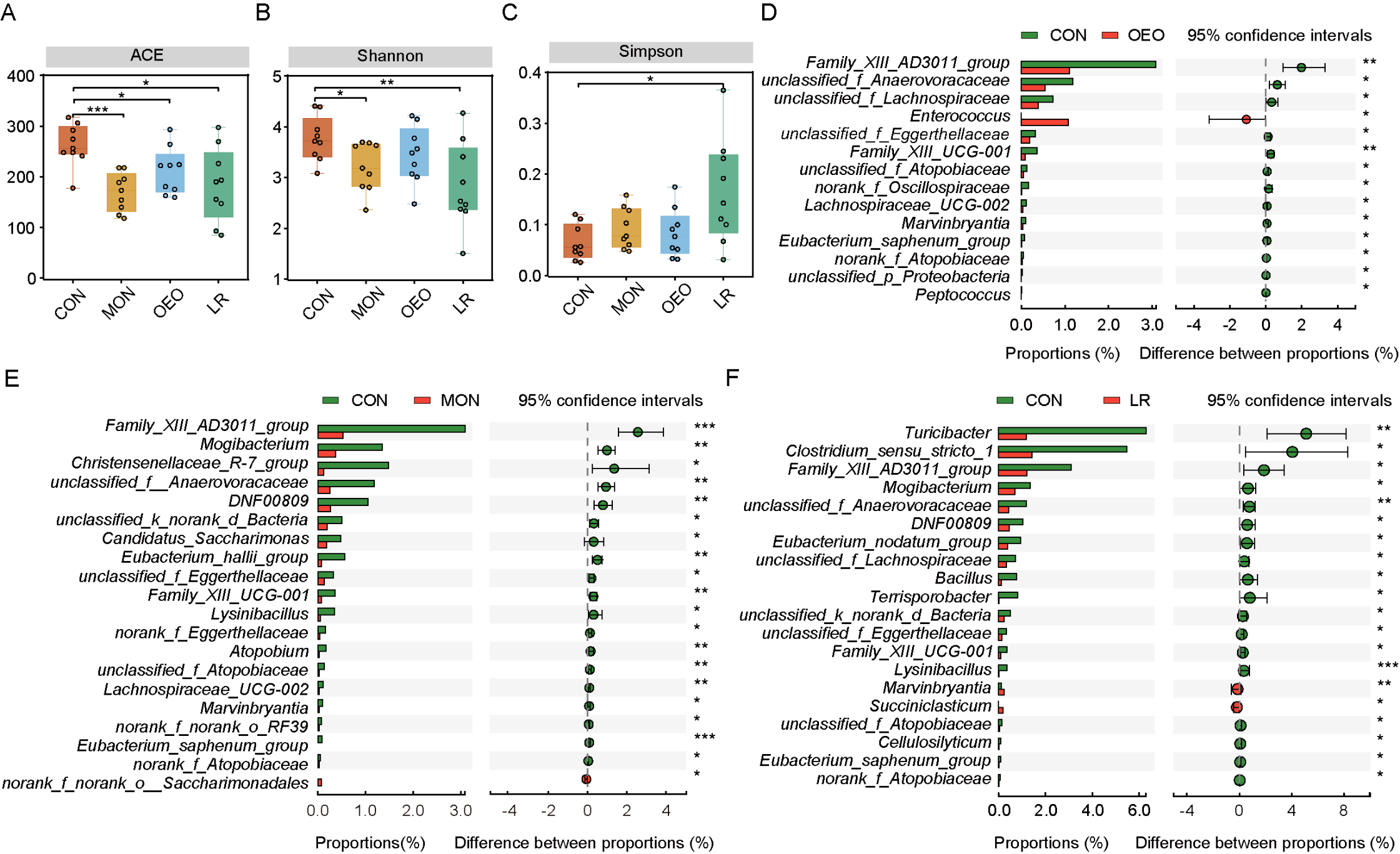


**Figure S1.** (A, B, C) Alpha diversity as presented by ACE, Shannon, Simpson index in the ileum contents of sheep among groups. (D, E, F) Extended error bar plot showing the bacteria at the genus level that had significant differences between the CON and trial groups. Positive differences indicate greater abundance of bacteria in the CON group, while negative differences indicate greater abundance in the trial group.


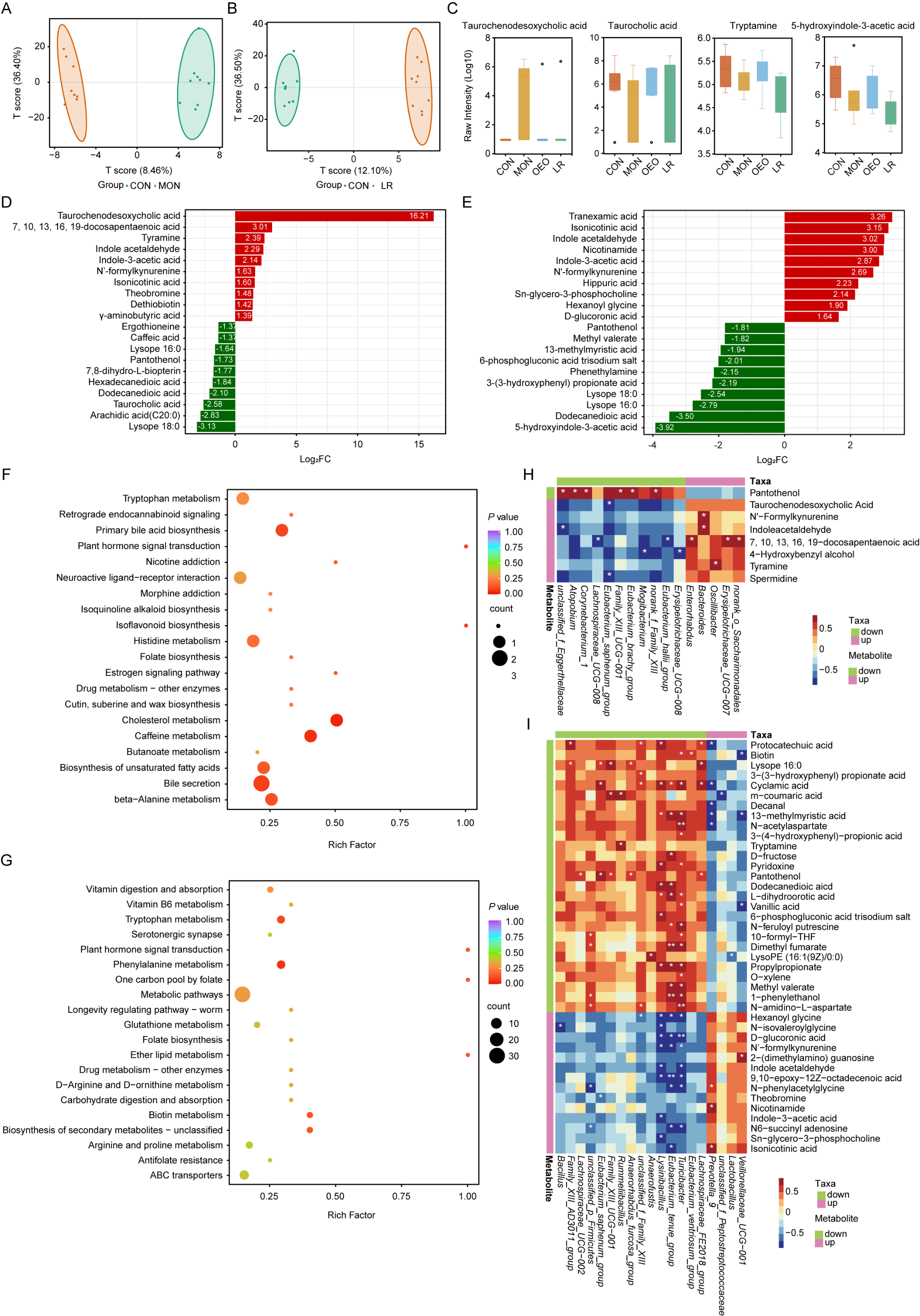


**Figure S2.** (A, B) OPLS-DA score plots showed significantly separated clusters between trial and CON group (CON vs MON, CON vs LR). (C) Box-plot of each significantly different metabolite peak area (Log10 transformed) among treatments. Data was presented as median and whiskers represented the Tukey. (D, E) Bar-chart showing the fold change of top 20 different metabolites (CON vs MON, CON vs LR). (F, G) The KEGG functional enrichment analysis of differential metabolites (CON vs MON, CON vs LR). (H, I) Correlation analysis of microbiota and metabolites in the ileal.

**Table S1.** Composition and nutrient levels of the basal diet, % of DM

| Ingredients | content |
| --- | --- |
| Corn straw silage | 17.18 |
| Corn straw | 8.86 |
| Corn | 44.31 |
| Bran | 3.69 |
| Concentrates | 8.86 |
| Soybean meal | 2.58 |
| Cottonseed meal | 3.69 |
| Rapeseed meal | 1.85 |
| Corncob | 4.43 |
| Malt sprout | 1.85 |
| Oil | 0.74 |
| Premix ^1)^ | 1.85 |
| NaCl | 0.07 |
| NaHCO_3_ | 0.04 |
| Total | 100 |
| Nutrient levels ^2)^ |  |
| DM | 88.30 |
| CP | 13.49 |
| DE/(MJ/kg) | 12.85 |
| NDF | 24.97 |
| Ca | 0.51 |
| P | 0.31 |

^1)^ The premix provided the following per kg of the diet: 100,000 IU of vitamin A, 800,00 IU of vitamin D, 550 IU of vitamin E, 1800 mg of Mn, 2100 mg of Zn, 1200 mg of Fe, 300 mg of Cu, 20 mg of I, 8 mg Se.

^2)^ Nutrient levels were measured value.

**Table S2.** Primers sequences used for real-time PCR

| Gene | Primers (sense/antisense 5'-3'） | Size (bp) | Reference/GenBank |
| --- | --- | --- | --- |
| ZO-1 | F: TGACGGTTGGTCTTTTGCTCT | 113 | XM_015101949 |
|  | R: CACAGTTTGCGCCAACAAGA |  |  |
| Occluding | F: CAGGGAATATCCACCTATCACTTCA | 102 | AJ313191.1 |
|  | R: TCAAGTTCTGCTTGTAGGCTCT |  |  |
| Claudin-1 | F: GTCTTTGGGGGCGTGATCTT | 150 | NM_001185016 |
|  | R: CCAGCCAATGAAGAGAGCCT |  |  |
| Mucin 1 | F: TCCACTGCCTCCCATCCTAA | 226 | XM_027976040 |
|  | R: ACACAGATCCTGGCCTGAAC |  |  |
| Mucin 2 | F: ACTGCCCTACACCAAGTTCG | 102 | XM_027959680 |
|  | R: GAGAGGCCGTTGTAGGACAC |  |  |
| Mucin 13 | F: ACCATTGCTGGCGTCGTTAT | 233 | XM_027962790 |
|  | R: TAGGGGCTTGTGTAGGGGTT |  |  |
| Mucin 20 | F: CCCTTGTCTTTCCCAAGTGC | 133 | XM_027956829 |
|  | R: CCAGTTTATCGCTGAGCTCCT |  |  |
| IFN-γ | F: GAACGGCAGCTCTGAGAAAC | 122 | NM_001009803 |
|  | R: TTGGCGACAGGTCATTCATCA |  |  |
| TNF-α | F: CTGCCTTGGCTCAGATGTGT | 220 | NM_001024860 |
|  | R: CTGACCAGTAGGGCGGTTAC |  |  |
| IL-6 | F: TGCAGTCCTCAAACGAGTGG | 110 | NM_001009392 |
|  | R: CCGCAGCTACTTCATCCGAA |  |  |
| TGF-β1 | F: GAAGTCTAGCTCGCACAGCA | 176 | NM_001009400 |
|  | R: TGAGGTAGCGCCAGGAATTG |  |  |
| GAPDH | F: AGAAACCTGCCAAGTATGATG | 196 | NM_00119039 |
|  | R: TCGTACCAGGAAATGAGCTTG |  |  |

F: Forward primer, R: Reverse primer; ZO-1: Zonula occludens 1; OCLN: Occluding; CLDN1: Claudin 1; MUC: Mucin; IFN-γ: Interferon-γ; IL-6: Interleukin-6; TNF-α: Tumor necrosis factor-α; TGF-β1: transforming growth factor-β1; GAPDH: Glyceraldehyde 3-phosphate dehydrogenase.

**Table S3.** Candidate ileal metabolites that differed between the control and treatment

| Compounds^a^ | VIP^b^ | Fold-Change | Log_2_FC | Type |
| --- | --- | --- | --- | --- |
| **CON vs MON** | | | | |
| Spermidine | 2.95 | 2.56 | 1.35 | up |
| Indole acetaldehyde | 2.77 | 4.90 | 2.29 | up |
| Indole-3-acetic acid | 2.70 | 4.42 | 2.14 | up |
| Punicic acid | 2.60 | 2.39 | 1.26 | up |
| N’-formylkynurenine | 2.47 | 3.09 | 1.63 | up |
| Taurochenodesoxycholic acid | 2.29 | 75963.15 | 16.21 | up |
| 4-hydroxybenzyl alcohol | 2.24 | 2.62 | 1.39 | up |
| Theobromine | 2.18 | 2.78 | 1.48 | up |
| Tyramine | 1.97 | 5.23 | 2.39 | up |
| 3-aminoisobutanoic acid | 1.84 | 2.19 | 1.13 | up |
| 7, 10, 13, 16, 19-docosapentaenoic acid | 1.65 | 8.04 | 3.01 | up |
| 2,6-diaminooimelic Acid | 1.48 | 2.22 | 1.15 | up |
| Dethiobiotin | 1.48 | 2.68 | 1.42 | up |
| γ-aminobutyric acid | 1.44 | 2.62 | 1.39 | up |
| Isonicotinic acid | 1.41 | 3.02 | 1.60 | up |
| 7,8-dihydro-L-biopterin | 2.46 | 0.29 | -1.77 | down |
| Pantothenol | 2.19 | 0.30 | -1.73 | down |
| Caffeic acid | 2.10 | 0.39 | -1.37 | down |
| 3-N-methyl-L-histidine | 1.77 | 0.48 | -1.05 | down |
| Lysope 16:0 | 1.73 | 0.32 | -1.64 | down |
| Lysope 18:0 | 1.64 | 0.11 | -3.13 | down |
| Xanthosine | 1.59 | 0.46 | -1.13 | down |
| Glycylphenylalanine | 1.57 | 0.42 | -1.25 | down |
| Ergothioneine | 1.50 | 0.39 | -1.37 | down |
| H-HomoArg-OH | 1.42 | 0.47 | -1.10 | down |
| Formononetin | 1.36 | 0.49 | -1.03 | down |
| 1-phenylethanol | 1.31 | 0.48 | -1.06 | down |
| Arachidic acid(C20:0) | 1.22 | 0.14 | -2.83 | down |
| Dodecanedioic acid | 1.21 | 0.23 | -2.10 | down |
| Taurocholic acid | 1.13 | 0.17 | -2.58 | down |
| Hexadecanedioic acid | 1.09 | 0.28 | -1.84 | down |
| **CON vs OEO** | | | | |
| Indole acetaldehyde | 2.66 | 5.43 | 2.44 | up |
| Indole-3-aetic acid | 2.60 | 5.07 | 2.34 | up |
| N’-formylkynurenine | 2.59 | 4.18 | 2.06 | up |
| 4-hydroxybenzyl alcohol | 2.33 | 2.44 | 1.29 | up |
| Theobromine | 2.21 | 3.24 | 1.70 | up |
| Tranexamic acid | 1.94 | 4.92 | 2.30 | up |
| L-ornithine | 1.68 | 2.21 | 1.14 | up |
| 2’-hydroxy-5’-methylacetophenone | 1.65 | 2.93 | 1.55 | up |
| 4-methylbenzoic acid | 1.62 | 2.39 | 1.26 | up |
| Purine | 1.53 | 2.44 | 1.29 | up |
| Thymidine | 1.49 | 2.55 | 1.35 | up |
| 6-methylnicotinamide | 1.49 | 2.15 | 1.11 | up |
| Deoxycytidine | 1.45 | 2.17 | 1.12 | up |
| Sn-glycero-3-phosphocholine | 1.45 | 3.71 | 1.89 | up |
| 2’-deoxyuridine | 1.38 | 2.88 | 1.53 | up |
| Cytosine | 1.35 | 2.28 | 1.19 | up |
| 5-methylcytosine | 1.34 | 2.88 | 1.52 | up |
| 2’-deoxyadenosine-5’-monophosphate | 1.34 | 2.22 | 1.15 | up |
| N-acetylphenylalanine | 1.30 | 2.23 | 1.16 | up |
| N-acetyl-L-leucine | 1.26 | 2.00 | 1.00 | up |
| Tyramine | 1.09 | 4.64 | 2.21 | up |
| m-coumaric acid | 2.59 | 0.35 | -1.53 | down |
| 2-(formylamino) benzoic acid | 2.57 | 0.38 | -1.39 | down |
| Dihydroactinidiolide | 2.35 | 0.32 | -1.62 | down |
| Hydroquinone | 2.26 | 0.37 | -1.45 | down |
| Indoxylsulfuric acid | 1.99 | 0.43 | -1.21 | down |
| Pantothenol | 1.95 | 0.34 | -1.55 | down |
| Kinic acid | 1.85 | 0.44 | -1.20 | down |
| Shikimic acid | 1.79 | 0.49 | -1.03 | down |
| Lysope 16:0 | 1.18 | 0.50 | -1.00 | down |
| Lysope 18:0 | 1.00 | 0.21 | -2.25 | down |
| **CON vs LR** | | | | |
| Indole acetaldehyde | 2.46 | 8.13 | 3.02 | up |
| N'-formylkynurenine | 2.31 | 6.45 | 2.69 | up |
| Indole-3-acetic acid | 2.28 | 7.29 | 2.87 | up |
| Tranexamic acid | 2.23 | 9.60 | 3.26 | up |
| Hexanoyl glycine | 2.15 | 3.74 | 1.90 | up |
| Theobromine | 1.92 | 2.67 | 1.42 | up |
| D-glucoronic acid | 1.83 | 3.12 | 1.64 | up |
| Spermidine | 1.80 | 2.00 | 1.00 | up |
| N-phenylacetylglycine | 1.73 | 2.80 | 1.48 | up |
| Isonicotinic acid | 1.71 | 8.87 | 3.15 | up |
| Sn-glycero-3-phosphocholine | 1.59 | 4.40 | 2.14 | up |
| N-isovaleroylglycine | 1.53 | 2.16 | 1.11 | up |
| N6-Succinyladenosine | 1.52 | 2.02 | 1.01 | up |
| 9,10-epoxy-12Z-octadecenoic acid | 1.52 | 2.53 | 1.34 | up |
| 2-(Dimethylamino)Guanosine | 1.46 | 2.98 | 1.57 | up |
| 9,10-dihydroxy-12Z-octadecenoic acid | 1.44 | 2.52 | 1.33 | up |
| Nicotinamide | 1.38 | 8.02 | 3.00 | up |
| L-cystine | 1.32 | 2.01 | 1.01 | up |
| Cytidine | 1.29 | 2.40 | 1.26 | up |
| 3’-aenylic acid | 1.26 | 2.14 | 1.10 | up |
| L-ornithine | 1.16 | 2.32 | 1.21 | up |
| Cytosine | 1.11 | 2.28 | 1.19 | up |
| Tryptophan betaine | 1.10 | 2.14 | 1.10 | up |
| Hippuric acid | 1.01 | 4.70 | 2.23 | up |
| N-acetylaspartate | 2.38 | 0.39 | -1.35 | down |
| Protocatechuic acid | 2.21 | 0.31 | -1.71 | down |
| 13-methylmyristic acid | 2.20 | 0.26 | -1.94 | down |
| Cyclamic acid | 2.18 | 0.29 | -1.79 | down |
| Lysope 16:0 | 2.08 | 0.14 | -2.79 | down |
| 5-hydroxyindole-3-acetic acid | 2.06 | 0.07 | -3.92 | down |
| Pyridoxine | 2.04 | 0.45 | -1.16 | down |
| N-amidino-L-aspartate | 2.01 | 0.37 | -1.42 | down |
| Lactose | 1.96 | 0.38 | -1.40 | down |
| 6-phosphogluconic acid trisodium salt | 1.95 | 0.25 | -2.01 | down |
| Pantothenol | 1.91 | 0.29 | -1.81 | down |
| Vanillic acid | 1.90 | 0.49 | -1.02 | down |
| 3-(4-hydroxyphenyl)-propionic acid | 1.87 | 0.44 | -1.18 | down |
| N-feruloyl putrescine | 1.86 | 0.47 | -1.10 | down |
| Decanal | 1.81 | 0.47 | -1.10 | down |
| Caffeic acid | 1.80 | 0.36 | -1.48 | down |
| 3-(3-hydroxyphenyl) propionate acid | 1.80 | 0.22 | -2.19 | down |
| L-dihydroorotic acid | 1.79 | 0.44 | -1.19 | down |
| Dodecanedioic acid | 1.79 | 0.09 | -3.50 | down |
| m-coumaric acid | 1.76 | 0.48 | -1.06 | down |
| O-xylene | 1.73 | 0.43 | -1.23 | down |
| Biotin | 1.69 | 0.44 | -1.17 | down |
| Hydroquinone | 1.68 | 0.49 | -1.04 | down |
| Propylpropionate | 1.66 | 0.30 | -1.75 | down |
| D-fructose | 1.66 | 0.37 | -1.45 | down |
| Vanillin | 1.64 | 0.50 | -1.01 | down |
| 10-formyl-THF | 1.55 | 0.43 | -1.23 | down |
| Methyl valerate | 1.51 | 0.28 | -1.82 | down |
| Tryptamine | 1.48 | 0.32 | -1.64 | down |
| Lysope 18:0 | 1.39 | 0.17 | -2.54 | down |
| Hydroxyphenyllactic acid | 1.39 | 0.48 | -1.06 | down |
| 7,8-dihydro-L-biopterin | 1.38 | 0.50 | -1.01 | down |
| Dethiobiotin | 1.31 | 0.44 | -1.18 | down |
| Deoxyadenosine | 1.30 | 0.36 | -1.48 | down |
| DL-stachydrine | 1.27 | 0.48 | -1.06 | down |
| Dimethyl fumarate | 1.23 | 0.45 | -1.14 | down |
| 1-phenylethanol | 1.21 | 0.44 | -1.17 | down |
| Phenethylamine | 1.19 | 0.23 | -2.15 | down |
| LysoPE (16:1(9Z)/0:0) | 1.18 | 0.38 | -1.38 | down |

^a^ CON, a basal diet; MON, a basal diet plus 0.5 g/head/d monensin; OEO, a basal diet with 52 mg/head/d oregano essential oil; LR, a basal diet supplemented with 10 g/head/d *lactobacillus reuteri*.

^b^ VIP, variable importance in the projection.
